# Engineered Production of Isobutanol from Sugarcane Trash Hydrolysates in *Pichia pastoris*

**DOI:** 10.3390/jof8080767

**Published:** 2022-07-25

**Authors:** Pornsiri Bumrungtham, Peerada Promdonkoy, Kanoknart Prabmark, Benjarat Bunterngsook, Katewadee Boonyapakron, Sutipa Tanapongpipat, Verawat Champreda, Weerawat Runguphan

**Affiliations:** National Center for Genetic Engineering and Biotechnology, 113 Thailand Science Park, Paholyothin Road, Klong 1, Klong Luang 12120, Pathum Thani, Thailand; pornsiri.bumr@gmail.com (P.B.); peerada.prom@biotec.or.th (P.P.); kanoknart.pra@ncr.nstda.or.th (K.P.); benjarat.bun@biotec.or.th (B.B.); katewadee.boo@biotec.or.th (K.B.); sutipa@biotec.or.th (S.T.); verawat@biotec.or.th (V.C.)

**Keywords:** isobutanol, *Pichia pastoris*, advanced biofuels, sugarcane trash hydrolysates, lignocellulosic biomass

## Abstract

Concerns over climate change have led to increased interest in renewable fuels in recent years. Microbial production of advanced fuels from renewable and readily available carbon sources has emerged as an attractive alternative to the traditional production of transportation fuels. Here, we engineered the yeast *Pichia pastoris*, an industrial powerhouse in heterologous enzyme production, to produce the advanced biofuel isobutanol from sugarcane trash hydrolysates. Our strategy involved overexpressing a heterologous xylose isomerase and the endogenous xylulokinase to enable the yeast to consume both C5 and C6 sugars in biomass. To enable the yeast to produce isobutanol, we then overexpressed the endogenous amino acid biosynthetic pathway and the 2-keto acid degradation pathway. The engineered strains produced isobutanol at a titer of up to 48.2 ± 1.7 mg/L directly from a minimal medium containing sugarcane trash hydrolysates as the sole carbon source. To our knowledge, this is the first demonstration of advanced biofuel production using agricultural waste-derived hydrolysates in the yeast *P. pastoris*. We envision that our work will pave the way for a scalable route to this advanced biofuel and further establish *P. pastoris* as a versatile production platform for fuels and high-value chemicals.

## 1. Introduction

Rising energy demands and growing concerns over climate change have led to significant interest in renewable fuels and chemicals [1,2,3]. In recent years, microbial production of advanced fuels via economically efficient bioprocesses has emerged as an attractive alternative to the traditional production of transportation fuels [4]. While microbial fermentation of ethanol has been pivotal in the transition to bio-based fuels, ethanol is not ideal as a gasoline replacement due to its poor physicochemical properties; ethanol has relatively low energy density, high hygroscopicity, and high vapor pressure [3]. On the other hand, branched-chain and higher alcohols, such as isobutanol and isoamyl alcohol, have higher energy density (at 90% and 110% of gasoline’s energy content, respectively) and are compatible with the existing storage and distribution infrastructures [5,6]. These superior properties, as well as their higher octane numbers compared with their straight-chain counterparts, make branched-chain and higher alcohols ideal gasoline substitutes for high-performance petrol engines.

Despite significant efforts in optimizing the natural producers of these alcohols, commercial production of the vast majority of these alcohols in native organisms such as several *Clostridium* species is not economically feasible at present [7]. Other disadvantages of using *Clostridium* species as a production host include their intolerance to oxygen, their slow growth, and their production of the byproducts acetone, butyrate, and ethanol. Therefore, the development of an efficient production platform in a non-native host for higher branched-chain alcohols is needed. 

In addition to product yield, the economic feasibility of a biofuel production process also depends on the choice of feedstocks [8]. Sugars derived from food crops are relatively expensive and can divert water and other scarce resources when demand for food and water is expected to surge [9]. Using agricultural waste-derived lignocellulosic feedstocks that do not compete for water and land with food would decrease costs and provide the most significant CO_2_ emission offsets [10,11]. Sugarcane trash is an abundant and underutilized biomass in sugar-producing countries worldwide [12]. It consists of approximately 15% of the total above-ground biomass at harvest, equivalent to 10–15 tons per hectare of dry matter. At present, only a fraction of sugarcane trash is converted to fuel at the sugar mill, while the rest is burned on site, creating dire pollution problems in haze and fine particles [13]. Using sugarcane trash as a lignocellulosic feedstock for biofuel production is therefore an attractive solution to both improve the economic feasibility of biofuels and alleviate the environmental problems caused by burning.

Here, we engineered the yeast *P. pastoris* (recently renamed *Komagataella phaffii*) to produce isobutanol from sugarcane trash hydrolysates. Our strategy involved overexpressing a heterologous xylose isomerase and the endogenous xylulokinase to enable the yeast to consume C5 and C6 sugars in the biomass hydrolysates. To enable the yeast to produce isobutanol, we overexpressed the endogenous L-valine biosynthetic pathway and the 2-keto acid degradation pathway. The engineered strains produced isobutanol at a titer of up to 48.2 ± 1.7 mg/L directly from a minimal medium containing sugarcane trash hydrolysates as the sole carbon source. We envision that our work will set the stage for a scalable route to this advanced biofuel. Moreover, our work further establishes *P. pastoris* as a versatile production platform for fuels and other chemicals.

## 2. Materials and Methods

### 2.1. Yeast Strain, Media, and Transformation 

The yeast strains used in this study were constructed from *P. pastoris* KM71 (Invitrogen) (Table 1). The plasmids used in this study were generated from the pGAPZA and pPIC3.5K vectors (Invitrogen) and pGAPHyg, pGAPHyg-PpIlv5-T2A1-PpIlv3, pGAPZ-LlkivD-T2A-ScADH7, and pGAPNeo-PpIlv6-T2A1-PpIlv2 plasmids [14]. Primers used for plasmid construction are listed in Supplementary Materials. Yeast transformation was performed as previously described using an electroporator with the following parameters: 1.5 kV, 25 μF, 200 Ω [15]. The integrative plasmids were linearized with the restriction enzyme AvrII before electroporation. Colony PCR and DNA sequencing were used to verify strain construction. *E. coli* was grown in a Luria–Bertani medium supplemented with ampicillin (at 100 μg/mL), hygromycin (at 100 μg/mL), kanamycin (at 50 μg/mL), or zeocin (at 25 μg/mL) when required. *P. pastoris* was grown in YPD medium (10 g/L yeast extract, 20 g/L Bacto Peptone, and 20 g/L glucose) supplemented with hygromycin (at 200 μg/mL), G418 (at 200 μg/mL), or zeocin (at 100 μg/mL) when required.

### 2.2. Plasmid Construction

Plasmid pGAPHyg-PspXI: The xylose isomerase from *Piromyces* sp. E2 (PspXI, GenBank accession number: AJ249909) was codon optimized for *P. pastoris* expression, synthesized, and provided as plasmid pCCI-PspXI by GenScript. The PspXI gene was amplified from pCCI-PspXI using primers PspXI-EcoRI-F and PspXI-NotI-R, and the amplicon was ligated to the EcoRI/NotI digested pGAPHyg to yield pGAPHyg-PspXI.

Plasmid pGAPHyg-LpXI: The xylose isomerase from *Lachnoclostridium phytofermentans* (LpXI; GenBank accession number: WP_012199251) was codon optimized for *P. pastoris* expression, synthesized, and provided as plasmid pCCI-LpXI by GenScript. The LpXI gene was amplified from pCCI-LpXI using primers LpXI-EcoRI-F and LpXI-NotI-R, and the amplicon was ligated to the EcoRI/NotI digested pGAPHyg to yield pGAPHyg-LpXI.

Plasmid pGAPZ-PpXKS: the xylulokinase from *Pichia pastoris* (PpXKS1; GenBank accession number: XM_002489890) was amplified from *P. pastoris* KM71 genomic DNA using primers PpXKS1-EcoRI-F and PpXKS1-NotI-R, and the amplicon was ligated to the EcoRI/NotI digested pGAPZ to yield pGAPZ-PpXKS.

Plasmid pGAPHyg-PspXI_pGCW14-PpXKS1: The promoter of GCW14 (GenBank accession number: XP_002490723) was amplified from *P. pastoris* KM71 genomic DNA using primers GCW14p-F and GCW14p-R. The PpXKS1 (GenBank accession number: XM_002489890) expression construct was amplified from pGAPZ-PpXKS using primers PpXKS1-F and PpXKS1-R. The two amplicons were joined together and ligated to the NsiI digested pGAPHyg-PspXI to yield pGAPHyg-PspXI_pGCW14-PpXKS1.

Plasmid pGAPHyg-LpXI_pGCW14-PpXKS1: The pGCW14-PpXKS1 cassette was amplified from pGAPHyg-PspXI_pGCW14-PpXKS1 using primers GCW14p-F and PpXKS1-R. The amplicon was ligated to the NsiI digested pGAPHyg-LpXI to yield pGAPHyg-LpXI_pGCW14-PpXKS1.

Plasmid pGAPZ-LlkivD-T2A-ScADH7_pGCW14-PpIlv5-T2A1-PpIlv3: The P_GCW14_ promoter was amplified from *P. pastoris* KM71 genomic DNA using primers GCW14p-2-F and GCW14p-2-R. The PpIlv5-T2A1-PpIlv3 construct was amplified from pGAPHyg-PpIlv5-T2A1-PpIlv3 using primers PpIlv5-F and AOX1t-R. The two amplicons were joined via overlap extension PCR and ligated to the BglII digested pGAPZ-LlkivD-T2A-ScADH7 to yield pGAPZ-LlkivD-T2A-ScADH7_pGCW14-PpIlv5-T2A1-PpIlv3.

### 2.3. Quantification of Isobutanol and Other Metabolites in Engineered Strains

Characterization of engineered *P. pastoris* strains was carried out in MGYH medium (13.6 g/L yeast nitrogen base without amino acids, 20 g/L glycerol, 0.1 M phosphate buffer pH 6.0, 0.4 mg/L D-biotin, 133.3 mg/L thiamine-hydrochloride, and 20 mg/L L-histidine) as previously reported with small modifications [14,16]. Briefly, engineered strains were precultured in 5-mL aliquots in MGYH medium overnight at 30 °C and used to inoculate 5 mL MGYH-glu medium (13.6 g/L yeast nitrogen base without amino acids, 20 g/L glucose, 0.1 M phosphate buffer pH 6.0, 0.4 mg/L D-biotin, 133.3 mg/L thiamine-hydrochloride, and 20 mg/L L-histidine) or, in the case of isobutanol production from sugarcane trash hydrolysates, MS_TH_YH medium (at a designated concentration of sugarcane trash hydrolysates) in 50 mL Corning tubes to achieve an initial optical density of 0.05 at 600 nm (OD_600_). The cultures were grown at 30 °C and 250 rpm in an orbital shaking incubator. Samples were taken to determine OD_600_, biomass, extracellular metabolites, and production of isobutanol.

The amount of isobutanol and other extracellular metabolites was determined using high-performance liquid chromatography (HPLC). Briefly, 1 mL of culture was centrifuged at 12,000 rpm for 5 min, and the supernatant was filtered through a 0.22 μm nylon syringe filter (Filtrex). The purified sample was then applied to an Agilent 1100 series HPLC equipped with an Aminex HPX-87H ion exchange column (Bio-Rad, Hercules, CA, USA). The LC program was performed using 5 mM H_2_SO_4_ as the solvent at a flow rate of 0.60 mL/min for 35 min. The column was maintained at 60 °C. All metabolites were detected with Agilent 1200 series DAD and RID detectors. 

### 2.4. Determination of the Engineered Strains’ Specific Growth Rates

Engineered strains were precultured in 5-mL aliquots in MGYH medium and grown overnight at 30 °C. The overnight cultures were diluted with fresh MGYH medium to obtain an OD_600_ of 2.0, and 25 uL aliquots of the diluted cultures were used to inoculate 975 uL of either MGYH-glu or MXYH medium (13.6 g/L yeast nitrogen base without amino acids, 20 g/L xylose, 0.1 M phosphate buffer pH 6.0, 0.4 mg/L D-biotin, 133.3 mg/L thiamine-hydrochloride, and 20 mg/L L-histidine) in a microflower plate. The cultures were cultivated in the BioLector microfermenter (m2p labs) at 30 °C at 1100 rpm for 96 h for real-time biomass measurement. The experiments were performed in biological triplicate. Scattered light measurements from the BioLector were converted to biomass (dry cell weight) by generating a standard curve using exponentially grown *P. pastoris* cells and comparing a dilution series on the BioLector and dry cell weight. 

### 2.5. Sugarcane Trash Pretreatment and Enzymatic Hydrolysis

Sugarcane trash was processed using a 20BVI pin mill (Shanghai UPG, Shanghai, China) and sieved to collect the particle with a diameter between 0.5 and 1.0 mm. The respective fraction was pretreated using the liquid hot water pretreatment method in a 7.5 L stainless steel high-pressure reactor (Parr Reactor 4848, Parr Instrument Company, Moline, IL, USA) with a biomass ratio to water of 1 g per 12 mL. The pretreatment was carried out at 180 °C with 400 rpm mixing speed and 0 min residence time. The reaction was subsequently cooled down for 2 h to room temperature. The solid fraction was collected and thoroughly washed with tap water until the pH reached 5.0. The sample was dried in an oven at 70 °C before undergoing enzymatic hydrolysis. 

The pretreated sugarcane trash was then hydrolyzed using Cellic^®^ CTec2 (Novozyme, Bagsvaerd, Denmark) in 800 mL working volume in a 2 L Erlenmeyer flask. The reaction contained 5% (*w*/*v*) solid loading of pretreated sugarcane trash and 20 mg protein/g substrate of Cellic^®^ CTec2 in 50 mM sodium acetate buffer pH 5.0. The reaction was incubated at 50 °C with a shaking speed of 200 rpm for 72 h. After that, the reaction was heat inactivated at 100 °C for 10 min. The supernatant was collected by centrifugation at 8000 rpm for 5 min at room temperature. The sugar profile and byproducts, including acetic acid, lactic acid, and levulinic acid, were analyzed using HPLC equipped with an Aminex HPX-87H ion exchange column. The LC program was performed using 5 mM H_2_SO_4_ as the solvent at a flow rate of 0.5 mL/min. The column was maintained at 65 °C. All metabolites were detected with Agilent 1200 series RID detectors. 

### 2.6. RNA Isolation and Transcript Quantification

Strains were precultured in 5-mL aliquots in MGYH medium overnight at 30 °C and used to inoculate 5 mL modified MS_TH_YH medium (at a designated concentration of sugarcane trash hydrolysates) in 50 mL Corning tubes to achieve an initial optical density of 0.05 at 600 nm (OD_600_). After 48 h at 30 °C, a 1-mL aliquot of each culture was collected and centrifuged for 5 min at 6000 rpm to collect the cell pellets. Total RNA was extracted using the QIAgen RNeasy Kit under the manufacturer’s protocol. Contaminating genomic DNA was removed from the RNA samples by DNaseI (Thermo Fisher Scientific, Waltham, MA, USA) digestion using the manufacturer′s protocol. The RNA quantity was analyzed using a NanoDrop ND-1000 spectrophotometer (NanoDrop Technologies, Wilmington, DE, USA), and samples were stored at −80 °C until RT-PCR analysis. cDNA was obtained using RevertAid Reverse Transcriptase (Thermo Fisher Scientific) using the manufacturer′s protocol. Relative expression levels of *PpIlv6*, *PpIlv2*, *PpIlv5*, *PpIlv3*, *LlkivD*, *ScADH7*, *PspXI*, *LpXI*, and *PpXKS* were quantified using the iQ SYBR Green Kit (Biorad) on CFX96 Touch Real-time PCR Detection System (Biorad). The ΔΔCt method was used to calculate the relative expression levels. Real-time PCR was performed in triplicates, and *PpACT1*, a gene that encodes actin, was used to normalize the amount of the total mRNA in all samples. Primers used for RT-PCR are listed in Supplementary Materials. 

## 3. Results and Discussion

### 3.1. Overexpression of Xylose Isomerase and Xylulokinase in P. pastoris Enables Cells to Grow in a Medium Containing Xylose as the Sole Carbon Source

**Figure 1:**
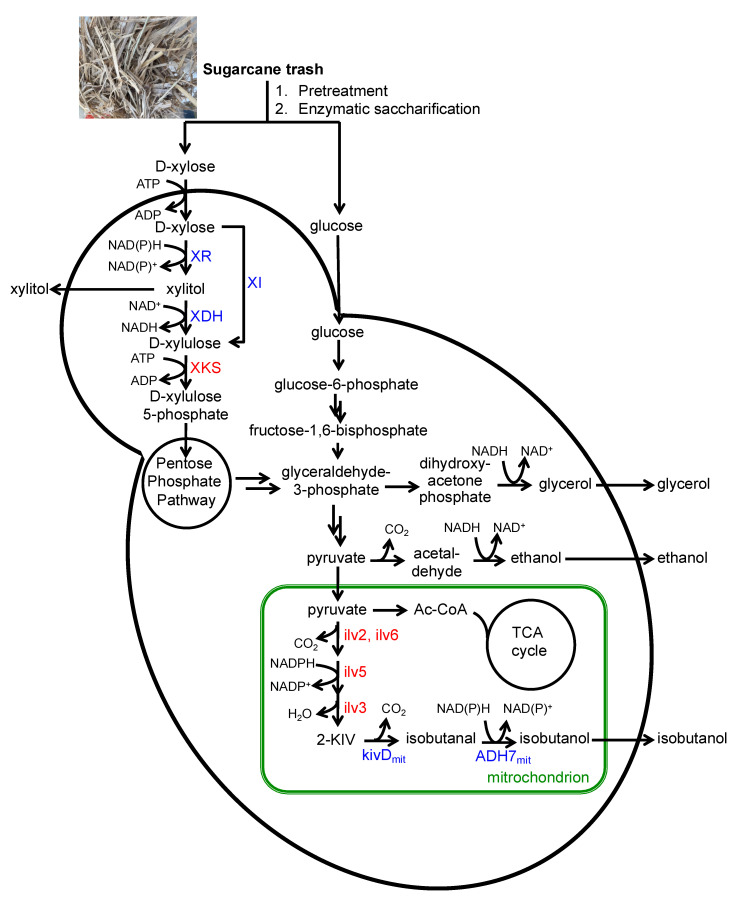
Engineered production of isobutanol from sugarcane trash hydrolysates in *P. pastoris*. XR xylose reductase, XDH xylitol dehydrogenase, XI xylose isomerase, XKS xylulokinase, Ilv2 acetolactate synthase, Ilv5 acetohydroxyacid reductoisomerase, Ilv3 dihydroxyacid dehydratase, kivD_mit_ mitochondria-targeted keto-acid decarboxylase, ADH7_mit_ mitochondria-targeted alcohol dehydrogenase. Enzymes highlighted in red are endogenous enzymes. Enzymes highlighted in blue are heterologous enzymes.

Since xylose is the second most abundant component of lignocellulosic biomass, having a bioprocess whereby the microbial host can efficiently consume xylose and produce advanced biofuels is of great interest. Therefore, we set out to engineer *P. pastoris* to efficiently utilize xylose as a carbon source and produce the advanced biofuel isobutanol (Figure 1). Two distinct pathways exist in xylose-fermenting organisms for the conversion of xylose to xylulose: the xylose reductase–xylitol dehydrogenase (XR–XDH) pathway in yeast and aerobic fungal species and the xylose–isomerase (XI) pathway in bacteria and anaerobic fungal species. We chose to overexpress the XI pathway in *P. pastoris* as this pathway neither requires any redox cofactor nor leads to the formation of the side product xylitol. Moreover, previous works have shown that xylose isomerases can be functionally expressed in *P. pastoris* [17]. For example, Li and coworkers co-expressed the *Orpinomyces* spp. XI and a gene encoding β-mannanase in *P. pastoris* GS115 leading to a strain that can produce the enzyme β-mannanase from a xylose-containing medium [18]. In addition to the XI pathway enzyme, we also overexpressed the endogenous xylulokinase (PpXKS) enzyme to facilitate the conversion of D-xylulose to D-xylulose 5-phosphate. 

**Figure 2:**
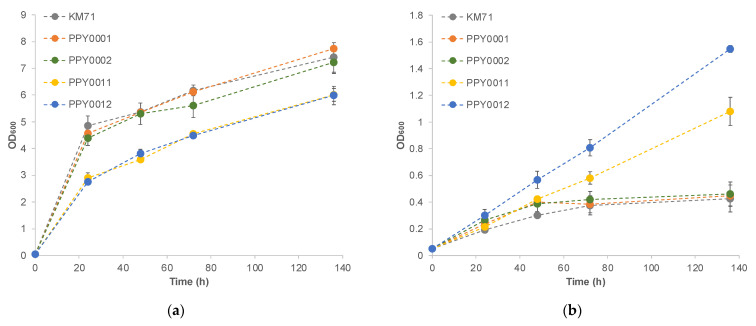
Growth profile of engineered *P. pastoris* strains in (**a**) MGYH-glu minimal medium containing 2% glucose as the sole carbon source and (**b**) MXYH minimal medium containing 2% xylose as the sole carbon source. Please note that the specific growth rates of the engineered strains were obtained using the BioLector microfermenter (m2p labs) and are listed in Table 1.

*P. pastoris* expressing PpXKS and either the bacterial or fungal XI grew significantly better than the control strain (the laboratory strain KM71) in a medium containing xylose as the sole carbon source (Table 1 and Figure 2). Strain PPY0011, which overexpresses the endogenous PpXKS and XI from the fungus *Piromyces* sp. E2 (PspXI), has a specific growth rate of 0.00267 ± 0.00005 h^−1^; a 3.8-fold improvement over KM71’s specific growth rate of 0.00070 ± 0.00008. Similarly, strain PPY0012, which overexpresses PpXKS and XI from the bacterium *Lachnoclostridium phytofermentans* (LpXI), has a specific growth rate of 0.00317 ± 0.00005 h^−1^; a 4.5-fold improvement over KM71′s value. Interestingly, strains PPY0001 and PPY0002, which overexpress the XI enzymes, but not PpXKS, exhibit only 10–19% improvement in specific growth rates over the control strain. These results suggest that the conversion of D-xylulose to D-xylulose 5-phosphate might be a potential bottleneck in the pathway and underscore the importance of XKS overexpression. Accumulatively, these results indicate that the heterologous expression of XI and overexpression of the endogenous XKS enabled the yeast to utilize xylose as a carbon source.

### 3.2. Engineered Strains with Overexpression of Xylose Isomerase and Xylulokinase Produce Higher Titer of Isobutanol in a Medium Containing Xylose and Glucose as a Mixed Carbon Source

Having demonstrated that *P. pastoris* can be engineered to utilize xylose as the sole carbon source by overexpressing a heterologous XI and the endogenous XKS, we next set out to introduce the isobutanol biosynthetic pathway into *P. pastoris*. In our earlier work, we engineered *P. pastoris* to produce isobutanol from glucose with a titer of up to 2.2 g/L [14]. This was achieved by exploiting the yeast’s endogenous amino acid biosynthetic pathway and diverting the amino acid intermediates to the 2-keto acid degradation pathway for higher alcohol production (Figure 1). Specifically, we overexpressed the endogenous L-valine biosynthetic pathway enzymes, PpIlv2, PpIlv6, PpIlv5, and PpIlv3 as well as the 2-keto acid degradation pathway enzymes ScADH7 from *Saccharomyces cerevisiae* and LlkivD from *Lactococcus lactis*. Expression of the latter two enzymes was targeted to the mitochondria to compartmentalize the isobutanol pathway enzymes into a single organelle, a technique that was used successfully to boost isobutanol production in the yeast *S. cerevisiae* by Avalos and coworkers and later in *P. pastoris* by our team [14,19]. Using these previous studies as a model for our strain design, we integrated the isobutanol pathway genes into both engineered strains PPY0011 and PPY0012, resulting in strains PPY0311 and PPY0312, respectively (Table 1). 

**Figure 3:**
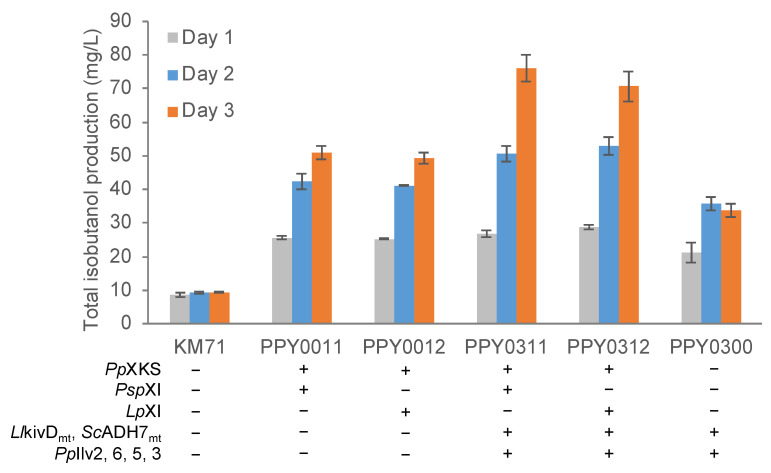
Total Isobutanol production of engineered *P. pastoris* in a mixed-carbon source medium. Engineered strains were precultured in 5-mL aliquots in MGYH minimal medium (2% glycerol) overnight and used to inoculate 5 mL yeast selective medium (2% total sugar; 1.70% glucose and 0.30% xylose) to achieve an initial optical density of 0.05 at 600 nm (OD_600_). The cultures were grown at 30 °C and 250 rpm in an orbital shaking incubator. Samples were taken at 24, 48, and 72 h for isobutanol quantification and OD_600_ measurement. Values are the mean of three biological replicates ± standard deviation (n = 3). Lp, *Lachnoclostridium phytofermentans*; Psp, *Piromyces* sp. E2; Pp, *Pichia pastoris*; Ll, *Lactococcus lactis*.

We next tested the ability of the engineered strains to produce isobutanol in a minimal medium containing a mixture of glucose and xylose at a ratio of 84.5:15.2 (2% total sugar; 1.70% glucose and 0.30% xylose). We set the ratio of the two carbon sources as such to emulate the ratio obtained in sugarcane bagasse hydrolysates obtained from our previous work [20]. The engineered strains PPY0311 and PPY0312 produced isobutanol at titers of 76.0 ± 4.0 and 70.6 ± 4.4 mg/L, respectively, a two-fold improvement over strain PPY0300 (33.8 ± 2.0 mg/L) that overexpresses only the isobutanol pathway genes but not the xylose utilization pathway genes (Figure 3 and Figure S2). Strains overexpressing only the xylose utilization pathway genes (PPY0011 and PPY0012) also produced isobutanol, though at lower titers compared with strains PPY0311 and PPY0312. It is important to note that, consistent with our previous findings [14], we also observed a small amount of isobutanol (9.4 ± 0.2 mg/L) being produced by the unengineered laboratory strain KM71. 

### 3.3. Preparation of Sugarcane Trash Hydrolysates 

Having demonstrated that strains PPY0311 and PPY0312 can produce isobutanol from a minimal medium containing a mixture of xylose and glucose, we turned to evaluate production from lignocellulosic agricultural wastes, specifically sugarcane trash. Sugarcane trash typically contains 40–60% cellulose content, 20–30% hemicellulose content, and the remaining 15–30% as lignin and ash [21]. We previously demonstrated that liquid hot water (LHW) pretreatment is an effective pretreatment method for sugarcane bagasse, resulting in high glucose and xylose yields [20,22,23,24]. LHW is a promising green pretreatment method using water as the sole reaction medium. Under high-pressure conditions, water exists as hydronium (H_3_O^+^) species and cleaves the side chain acetic acid from hemicellulose. This results in the generation of acetic acid, which then autocatalyzes hydrolysis of the hemicellulose fraction and also partial removal of the associated lignin. For this work, we used liquid hot water pretreatment at 180 °C, followed by enzymatic hydrolysis of the pretreated biomass using the commercial Cellic^®^ CTec2 enzyme. The resulting hydrolysates contained 30.22 ± 0.18 g/L total sugar content (22.66 ± 0.14 g/L glucose and 7.55 ± 0.11 g/L xylose), which is equivalent to 92.39% glucose recovery and 69.92% xylose recovery based on cellulose and hemicellulose available in pretreated sugarcane trash. In addition to sugars, the hydrolysates also contained organic acids (0.59 ± 0.00 g/L lactic acid, 4.84 ± 0.01 g/L acetic acid, and 0.02 ± 0.00 g/L levulinic acid) as pretreatment byproducts.

### 3.4. Isobutanol Production from Sugarcane Trash Hydrolysates 

Encouraged by the results from the experiments using the mixed carbon source medium, we turned to test the engineered strains’ ability to produce isobutanol directly from sugarcane trash hydrolysates. While production of advanced biofuels and chemicals from lignocellulosic hydrolysates using model organisms such as the budding yeast *S. cerevisiae* and the bacterium *E. coli* is well established, similar work using the yeast *P. pastoris* remains scarce [11,25]. Indeed, we found only two previous reports of engineering *P. pastoris* to produce bulk enzymes from biomass hydrolysates and just one report using the yeast as a whole-cell biocatalyst for converting xylose to xylitol [18,26,27]. Li and coworkers set the stage by engineering *P. pastoris* to be able to consume xylose—the first study to do so—and produce β-mannanase, a bulk enzyme used in animal feed, textile, and other industries [18]. More recently, Bankefa and coworkers engineered *P. pastoris* to produce the enzymes β-galactosidase and β-mannanase [26]. For chemical production, Louie and coworkers expressed a xylitol dehydrogenase enzyme (along with a glucose dehydrogenase to regenerate the cofactor NAD(P)H) in *P. pastoris*. The recombinant strain was then used as a biocatalyst to convert xylose into xylitol [27].

**Figure 4:**
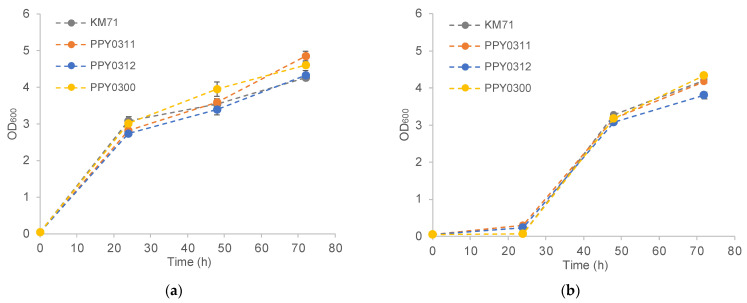
Growth profile of engineered *P. pastoris* strains in minimal medium containing different concentrations of sugarcane trash hydrolysates as the sole carbon source. (**a**) Growth in minimal medium with sugarcane trash hydrolysates with a total sugar content of 1.5%; (**b**) growth in minimal medium with sugarcane trash hydrolysates with a total sugar content of 2.0%.

The sugarcane trash hydrolysates obtained in this study from the liquid hot water pretreatment followed by enzymatic hydrolysis contained several organic acids (0.59 ± 0.00 g/L lactic acid, 4.84 ± 0.01 g/L acetic acid, and 0.02 ± 0.00 g/L levulinic acid) as pretreatment byproducts. A large body of work indicates that organic acids (along with other pretreatment inhibitors) can adversely affect yeast’s growth and even inhibit it completely at high concentrations [28,29]. As such, an optimal concentration of sugarcane trash hydrolysate should be empirically determined; not too low as to result in low isobutanol titer but also not too high to result in growth inhibition. While many strategies exist to detoxify the inhibitory compounds in lignocellulosic hydrolysates, such as the application of microbial or enzymatic detoxification, they tend to add cost and time to the bioprocess [30]. Therefore, in this work, we opted to use the sugarcane trash hydrolysates without an additional detoxification step. We screened two concentrations (1.5% and 2.0% total sugar concentration) of sugarcane trash hydrolysates to identify the optimal concentration for isobutanol production and yeast growth (Figure 4). Overall, the engineered strains exhibit robust growth in a medium containing sugarcane trash hydrolysates at 1.5% total sugar concentration. However, at 2.0% total sugar concentration, all tested strains displayed a prolonged lag phase, commonly observed when cells undergo a genomic adaptation process in response to the inhibitors [31]. After the lag phase, all tested strains achieved similar growth at the 72-h time point compared with the strains grown in the lower concentration of sugarcane trash hydrolysates.

**Figure 5:**
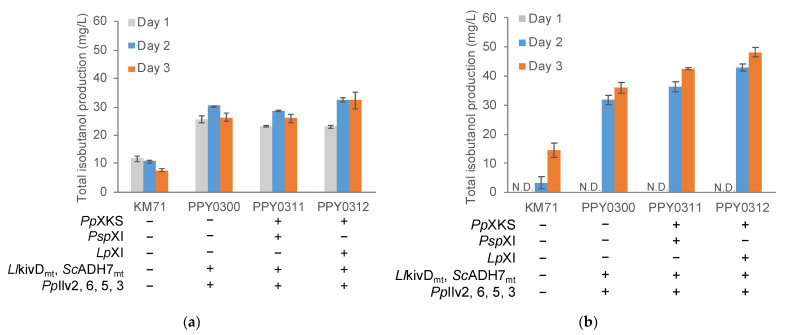
Total isobutanol production of engineered *P. pastoris* in a sugarcane trash medium. (**a**) Isobutanol production in minimal medium with sugarcane trash hydrolysates with a total sugar content of 1.5%; (**b**) isobutanol production in minimal medium with sugarcane trash hydrolysates with a total sugar content of 2%. N.D.—not detected.

We tested the ability of the engineered strains to produce isobutanol in a minimal medium containing sugarcane trash hydrolysates as the sole carbon source (Figure 5). We used the two screened sugarcane trash hydrolysate concentrations (total sugar concentrations of 1.5% and 2.0%). Gratifyingly, we observed isobutanol production in strains overexpressing the xylose utilization pathway genes and isobutanol pathway genes at both sugarcane trash hydrolysate concentrations. Despite the prolonged lag phase observed in the higher concentration of sugarcane trash hydrolysates resulting in an undetectable amount of isobutanol on Day 1, all tested strains produced higher amounts of isobutanol on subsequent days. In particular, strain PPY0312 produced isobutanol at 48.2 ± 1.7 mg/L in sugarcane trash hydrolysate medium with a total sugar content of 2%. The observed titer is a 34% improvement over the level produced by strain PPY0300 (36.1 ± 1.8 mg/L), which lacks the xylose utilization pathway, and a 230% improvement over the laboratory strain KM71 (14.6 ± 2.4 mg/L). Similarly, strain PPY0311 produced isobutanol at 42.6 ± 0.4 mg/L, an 18% and 191% improvement over strain PPY0300 and KM71, respectively, in the same medium. 

**Figure 6:**
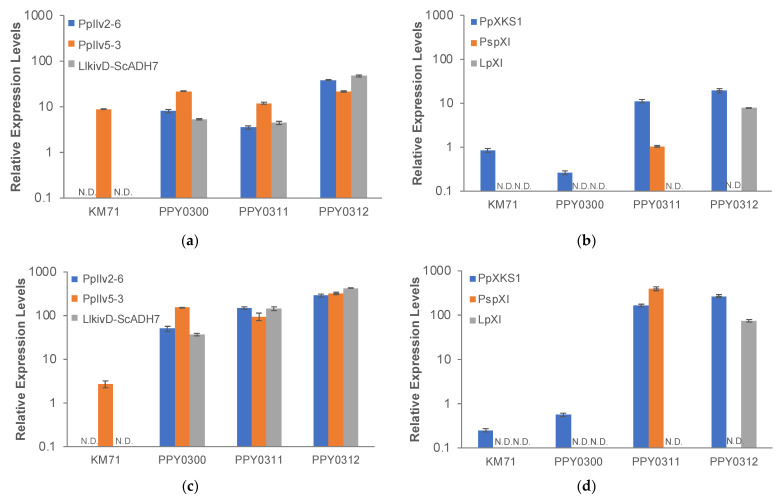
RT-PCR analysis of xylose utilization and isobutanol pathway genes of engineered strains in a sugarcane trash medium. (**a**) Expression of *PpIlv2_codon optimized*, *PpIlv6_codon optimized*, *PpIlv5*, *PpIlv3*, *LlkivD*, and *ScADH7* of engineered strains in minimal medium with sugarcane trash hydrolysates with a total sugar content of 1.5%; (**b**) expression of *PpXKS*, *PspXI,* and *LpXI* of engineered strains in minimal medium with sugarcane trash hydrolysates with a total sugar content of 1.5%; (**c**) expression of *PpIlv2_codon optimized*, *PpIlv6_codon optimized*, *PpIlv5*, *PpIlv3*, *LlkivD,* and *ScADH7* of engineered strains in minimal medium with sugarcane trash hydrolysates with a total sugar content of 2.0%; (**d**) expression of *PpXKS*, *PspXI,* and *LpXI* of engineered strains in minimal medium with sugarcane trash hydrolysates with a total sugar content of 2.0%.

To verify that the increase in isobutanol production in sugarcane trash hydrolysate medium with a total sugar content of 2% correlated with increased expression levels of the pathway genes, we performed RT-PCR in the three engineered strains, PPY0311, PPY0312, PPY0300, as well as the laboratory strain KM71 (Figure 6). The expression levels of the isobutanol pathway genes (*Pp*Ilv2, *Pp*Ilv5, *Pp*Ilv3, *Pp*Ilv6, *LlkivD*, and *ScADH7*) and the xylose utilization genes (*PpXKS* and *PspXI* (for PPY0311) or *LpXI (*for PPY0312)) were higher in sugarcane trash hydrolysate medium with a total sugar content of 2% compared with the levels observed at lower sugarcane trash hydrolysate concentration (Figure 6a vs. Figure 6c and Figure 6b vs. Figure 6d). Our results are consistent with previous findings and indicate that the total sugar concentration in the medium can impact the strengths of P_GAP_ and P_GCW14_ promoters, which were used to drive the expression of the isobutanol pathway genes and the xylose utilization pathway genes [14]. 

Notably, the isobutanol titers produced by our engineered strains PPY0311 and PPY0312 using sugarcane trash hydrolysates remain well below the grams/liter level obtained when using pure glucose as the carbon source described in our earlier work [14,15]. While product titers can typically be improved by increasing the amount of carbon source in the fermentation medium (though only to a certain point and product yield might be adversely affected) [32], in our case, however, increasing the concentration of sugarcane trash hydrolysates also means letting in higher concentrations of the toxic pretreatment inhibitors which would be detrimental to yeast growth. As such, we posit that our engineered strains would most benefit from increased tolerance to pretreatment inhibitors. Several strategies exist to achieve this. For example, strains PPY0311 and PPY0312 can be subjected to adaptive laboratory evolution in a medium with increasing concentrations of sugarcane trash hydrolysates to obtain strains that better tolerate pretreatment inhibitors. This strategy has been successfully applied to improve the fermentation performance of other microbial hosts and should apply to *P. pastoris* as well [33].

In addition to increasing the strain’s tolerance to pretreatment inhibitors, other strategies to boost the consumption of the sugarcane trash hydrolysates and isobutanol production include fine tuning the expression levels of the xylose utilization pathway and isobutanol pathway genes. This can be done by varying the copy number of the introduced genes either by using iterative integration of the gene expression construct or by using an episomal plasmid-based expression system [34,35]. Alternatively, one can vary the promoter strength driving the expression of these genes. Recent works have identified several constitutive promoters of various strengths that can drive gene expression in *P. pastoris* [36,37,38]. Finally, one can also use metabolic flux analysis to identify bottlenecks in the pathway and engineer the strain further to relieve these bottlenecks [39,40].

## 4. Conclusions

In this study, we engineered the yeast *P. pastoris* to produce isobutanol from sugarcane trash hydrolysates. Heterologous expression of xylose isomerase and overexpression of the endogenous xylulokinase enabled the yeast to grow in a medium containing xylose as the sole carbon source. Further introduction of the isobutanol pathway genes comprising the valine biosynthetic pathway genes as well as the 2-keto acid degradation pathway genes resulted in yeast strains PPY0311 and PPY0312 that can produce isobutanol from a mixed-carbon source medium. Moreover, PPY0311 and PPY0312 produced isobutanol at a titer of up to 48.2 ± 1.7 mg/L directly from a minimal medium containing sugarcane trash hydrolysates as the sole carbon source. To our knowledge, this is the first demonstration of advanced biofuel production using agricultural waste-derived hydrolysates in the yeast *P. pastoris*. We envision that our work will pave the way for a scalable route to this advanced biofuel and further establish *P. pastoris* as a versatile production platform for fuels and other chemicals.

## Figures and Tables

**Table 1:** Engineered strains generated in this study.

Strain Name	Overexpressed Genes ^1^	Specific Growth Rates in MGYH-glu (h^−1^)	Specific Growth Rates in MXYH(h^−1^)	References
KM71	None	0.07583 ± 0.00066	0.00070 ± 0.00008	Invitrogen
PPY0001	*PspXI*	0.07790 ± 0.00099	0.00077 ± 0.00005	This paper
PPY0002	*LpXI*	0.07730 ± 0.00108	0.00083 ± 0.00005	This paper
PPY0011	*PspXI*, *PpXKS*	0.05330 ± 0.00106	0.00267 ± 0.00005	This paper
PPY0012	*LpXI*, *PpXKS*	0.06050 ± 0.00128	0.00317 ± 0.00005	This paper
PPY0111	*PspXI*, *PpXKS*, *PpIlv2*, *PpIlv6*	0.05747 ± 0.00094	0.00257 ± 0.00008	This paper
PPY0112	*LpXI*, *PpXKS*, *PpIlv2*, *PpIlv6*	0.05900 ± 0.00179	0.00260 ± 0.00008	This paper
PPY0211	*PspXI*, *PpXKS*, *PpIlv5*, *PpIlv3*, *LlkivD*, *ScADH7*	0.04963 ± 0.00264	0.00193 ± 0.00017	This paper
PPY0212	*LpXI*, *PpXKS*, *PpIlv5*, *PpIlv3*, *LlkivD*, *ScADH7*	0.05813 ± 0.00133	0.00287 ± 0.00005	This paper
PPY0311	*PspXI*, *PpXKS*, *PpIlv2*, *PpIlv6*, *PpIlv5*, *PpIlv3*, *LlkivD*, *ScADH7*	0.05097 ± 0.00301	0.00260 ± 0.00008	This paper
PPY0312	*LpXI*, *PpXKS*, *PpIlv2*, *PpIlv6*, *PpIlv5*, *PpIlv3*, *LlkivD*, *ScADH7*	0.06153 ± 0.00111	0.00280 ± 0.00014	This paper
PPY0100	*PpIlv2*, *PpIlv6*	0.07303 ± 0.00264	0.00067 ± 0.00005	This paper
PPY0200	*PpIlv5*, *PpIlv3*, *LlkivD*, *ScADH7*	0.06573 ± 0.00146	0.00073 ± 0.00005	This paper
PPY0300	*PpIlv2*, *PpIlv6*, *PpIlv5*, *PpIlv3*, *LlkivD*, *ScADH7*	0.06330 ± 0.00206	0.00083 ± 0.00005	This paper

^1^ *PspXI*, *LpXI*, *LlkivD*, *PpIlv2,* and *PpIlv6* were codon optimized for *P. pastoris* expression.

## Data Availability

Data sharing is not applicable to this article.

## Supplementary Materials

The following supporting information can be downloaded at: https://www.mdpi.com/article/10.3390/jof8080767/s1, Table S1: Primers used in this study. Figure S1: Plasmid maps for plasmids used in this study. Figure S2: Growth of engineered *P. pastoris* in a mixed-carbon source medium.

## References

[1] Fortman J.L., Chhabra S., Mukhopadhyay A., Chou H., Lee T.S., Steen E., Keasling J.D. (2008). Biofuel Alternatives to Ethanol: Pumping the Microbial Well. Trends Biotechnol..

[2] Kung Y., Runguphan W., Keasling J.D. (2012). From Fields to Fuels: Recent Advances in the Microbial Production of Biofuels. ACS Synth. Biol..

[3] Peralta-Yahya P.P., Zhang F., del Cardayre S.B., Keasling J.D. (2012). Microbial Engineering for the Production of Advanced Biofuels. Nature.

[4] Patel A., Shah A.R. (2021). Integrated Lignocellulosic Biorefinery: Gateway for Production of Second Generation Ethanol and Value Added Products. J. Bioresour. Bioprod..

[5] Lamsen E.N., Atsumi S. (2012). Recent Progress in Synthetic Biology for Microbial Production of C3–C10 Alcohols. Front. Microbiol..

[6] Runguphan W., Sae-Tang K., Tanapongpipat S. (2021). Recent Advances in the Microbial Production of Isopentanol (3-Methyl-1-Butanol). World J. Microbiol. Biotechnol..

[7] Lütke-Eversloh T., Bahl H. (2011). Metabolic Engineering of Clostridium Acetobutylicum: Recent Advances to Improve Butanol Production. Curr. Opin. Biotechnol..

[8] Scown C.D., Baral N.R., Yang M., Vora N., Huntington T. (2021). Technoeconomic Analysis for Biofuels and Bioproducts. Curr. Opin. Biotechnol..

[9] UNESCO (2020). The United Nations World Water Development Report 2020: Water and Climate Change.

[10] Caspeta L., Nielsen J. (2013). Economic and Environmental Impacts of Microbial Biodiesel. Nat. Biotechnol..

[11] Su Y., Zhang W., Zhang A., Shao W. (2020). Biorefinery: The Production of Isobutanol from Biomass Feedstocks. Appl. Sci..

[12] Chandel A.K., da Silva S.S., Carvalho W., Singh O.V. (2012). Sugarcane Bagasse and Leaves: Foreseeable Biomass of Biofuel and Bio-Products. J. Chem. Technol. Biotechnol..

[13] Arbex M.A., Martins L.C., De Oliveira R.C., Pereira L.A.A., Arbex F.F., Cançado J.E.D., Saldiva P.H.N., Braga A.L.F. (2007). Air Pollution from Biomass Burning and Asthma Hospital Admissions in a Sugar Cane Plantation Area in Brazil. J. Epidemiol. Community Health.

[14] Siripong W., Wolf P., Kusumoputri T.P., Downes J.J., Kocharin K., Tanapongpipat S., Runguphan W. (2018). Metabolic Engineering of Pichia Pastoris for Production of Isobutanol and Isobutyl Acetate. Biotechnol. Biofuels.

[15] Siripong W., Angela C., Tanapongpipat S., Runguphan W. (2020). Metabolic Engineering of Pichia Pastoris for Production of Isopentanol (3-Methyl-1-Butanol). Enzyme Microb. Technol..

[16] Promdonkoy P., Mhuantong W., Champreda V., Tanapongpipat S., Runguphan W. (2020). Improvement in D-Xylose Utilization and Isobutanol Production in S. Cerevisiae by Adaptive Laboratory Evolution and Rational Engineering. J. Ind. Microbiol. Biotechnol..

[17] Ruchala J., Sibirny A.A. (2021). Pentose Metabolism and Conversion to Biofuels and High-Value Chemicals in Yeasts. FEMS Microbiol. Rev..

[18] Li P., Sun H., Chen Z., Li Y., Zhu T. (2015). Construction of Efficient Xylose Utilizing Pichia Pastoris for Industrial Enzyme Production. Microb. Cell Fact..

[19] Avalos J.L., Fink G.R., Stephanopoulos G. (2013). Compartmentalization of Metabolic Pathways in Yeast Mitochondria Improves the Production of Branched-Chain Alcohols. Nat. Biotechnol..

[20] Bunterngsook B., Laothanachareon T., Chotirotsukon C., Inoue H., Fujii T., Hoshino T., Roongsawang N., Kuboon S., Kraithong W., Techanan W. (2018). Development of Tailor-Made Synergistic Cellulolytic Enzyme System for Saccharification of Steam Exploded Sugarcane Bagasse. J. Biosci. Bioeng..

[21] Singh P., Suman A., Tiwari P., Arya N., Gaur A., Shrivastava A.K. (2008). Biological Pretreatment of Sugarcane Trash for Its Conversion to Fermentable Sugars. World J. Microbiol. Biotechnol..

[22] Imman S., Kreetachat T., Khongchamnan P., Laosiripojana N., Champreda V., Suwannahong K., Sakulthaew C., Chokejaroenrat C., Suriyachai N. (2021). Optimization of Sugar Recovery from Pineapple Leaves by Acid-Catalyzed Liquid Hot Water Pretreatment for Bioethanol Production. Energy Rep..

[23] Imman S., Laosiripojana N., Champreda V. (2018). Effects of Liquid Hot Water Pretreatment on Enzymatic Hydrolysis and Physicochemical Changes of Corncobs. Appl. Biochem. Biotechnol..

[24] Khongchamnan P., Suriyachai N., Kreetachat T., Laosiripojana N., Weerasai K., Champreda V., Suwannahong K., Sakulthaew C., Chokejaroenrat C., Imman S. (2022). Optimization of Liquid Hot Water Pretreatment and Fermentation for Ethanol Production from Sugarcane Bagasse Using Saccharomyces Cerevisiae. Catalysts.

[25] Zhu T., Sun H., Wang M., Li Y. (2019). Pichia Pastoris as a Versatile Cell Factory for the Production of Industrial Enzymes and Chemicals: Current Status and Future Perspectives. Biotechnol. J..

[26] Bankefa O.E., Samuel-Osamoka F.C., Oladeji S.J. (2022). Improved Enzyme Production on Corncob Hydrolysate by a Xylose-Evolved Pichia Pastoris Cell Factory. J. Food Sci. Technol..

[27] Louie T.M., Louie K., DenHartog S., Gopishetty S., Subramanian M., Arnold M., Das S. (2021). Production of Bio-Xylitol from d-Xylose by an Engineered Pichia Pastoris Expressing a Recombinant Xylose Reductase Did Not Require Any Auxiliary Substrate as Electron Donor. Microb. Cell Fact..

[28] Jönsson L.J., Martín C. (2016). Pretreatment of Lignocellulose: Formation of Inhibitory by-Products and Strategies for Minimizing Their Effects. Bioresour. Technol..

[29] Paes B.G., Steindorff A.S., Formighieri E.F., Pereira I.S., Almeida J.R.M. (2021). Physiological Characterization and Transcriptome Analysis of Pichia Pastoris Reveals Its Response to Lignocellulose-Derived Inhibitors. AMB Express.

[30] Parawira W., Tekere M. (2011). Biotechnological Strategies to Overcome Inhibitors in Lignocellulose Hydrolysates for Ethanol Production: Review. Crit. Rev. Biotechnol..

[31] Liu Z.L. (2006). Genomic Adaptation of Ethanologenic Yeast to Biomass Conversion Inhibitors. Appl. Microbiol. Biotechnol..

[32] Pothakos V., Debeer N., Debonne I., Rodriguez A., Starr J.N., Anderson T. (2018). Fermentation Titer Optimization and Impact on Energy and Water Consumption during Downstream Processing. Chem. Eng. Technol..

[33] Menegon Y.A., Gross J., Jacobus A.P. (2022). How Adaptive Laboratory Evolution Can Boost Yeast Tolerance to Lignocellulosic Hydrolyses. Curr. Genet..

[34] Gao S., Tong Y., Zhu L., Ge M., Zhang Y., Chen D., Jiang Y., Yang S. (2017). Iterative Integration of Multiple-Copy Pathway Genes in Yarrowia Lipolytica for Heterologous β-Carotene Production. Metab. Eng..

[35] Camattari A., Goh A., Yip L.Y., Meng Tan A.H., Ng S.W., Tran A., Liu G., Liachko I., Dunham M.J., Rancati G. (2016). Characterization of a PanARS-Based Episomal Vector in the Methylotrophic Yeast Pichia Pastoris for Recombinant Protein Production and Synthetic Biology Applications. Microb. Cell Fact..

[36] Xu N., Zhu J., Zhu Q., Xing Y., Cai M., Jiang T., Zhou M., Zhang Y. (2018). Identification and Characterization of Novel Promoters for Recombinant Protein Production in Yeast Pichia Pastoris. Yeast.

[37] Prattipati M., Ramakrishnan K., Sankaranarayanan M. (2020). Pichia Pastoris Protein Disulfide Isomerase (PDI1) Promoter for Heterologous Protein Production and Its Sequence Characterization. Enzyme Microb. Technol..

[38] Naseri G., Prause K., Hamdo H.H., Arenz C. (2021). Artificial Transcription Factors for Tuneable Gene Expression in Pichia Pastoris. Front. Bioeng. Biotechnol..

[39] Nie X., Hua Q., Xu P., Yang C. (2020). Biological Insights into Non-Model Microbial Hosts through Stable-Isotope Metabolic Flux Analysis. Curr. Opin. Biotechnol..

[40] Jordà J., Jouhten P., Cámara E., Maaheimo H., Albiol J., Ferrer P. (2012). Metabolic Flux Profiling of Recombinant Protein Secreting Pichia Pastoris Growing on Glucose:Methanol Mixtures. Microb. Cell Fact..

