# Supplementary material for: Engineered Production of Isobutanol from Sugarcane Trash Hydrolysates in Pichia pastoris
Source: J Fungi (Basel). 2022 Jul 25;8(8):767. doi: 10.3390/jof8080767 (PMC9330720; doi:10.3390/jof8080767)
Supplement: Supplementary file 1 [file jof-08-00767-s001.zip › jof-1805453-supplementary.pdf]

Supplementary Materials for Engineered Production of Isobutanol from Sugarcane Trash Hydrolysates in *Pichia pastoris*

Pornsiri Bumrungtham, Peerada Promdonkoy, Kanoknart Prabmark, Benjarat Bunterngsook, Katewadee Boonyapakron, Sutipa Tanapongpipat, Verawat Champreda and Weerawat Runguphan *

National Center for Genetic Engineering and Biotechnology, 113 Thailand Science Park, Paholyothin Road, Klong 1, Klong Luang, Pathum Thani 12120, Thailand

Corresponding Author

Tel.: +66-02-564-6700, Fax: +66-02-564-6701

Address: National Center for Genetic Engineering and Biotechnology, 113 Thailand Science Park, Paholyothin Road, Klong 1, Klong Luang, Pathum Thani 12120, Thailand

E-mail: weerawat.run@biotec.or.th

Table S1. Primers used in this study

Primer Name	Sequence (5′ to 3′)
PspXI-EcoRI-F	ATAGAATTCGAAACGATGGCTAAGGAA
PspXI-NotI-R	ATAGCGGCCGCTTATTGATACATAGCAACA
LpXI-EcoRI-F	ATAGAATTCGAAACGATGAAGAACTAC
LpXI-NotI-R	ATAGCGGCCGCTTATCTAAACAAAATATTG
PpXKS1-EcoRI-F	ATAGAATTCGAAACGATGGTTACCAAAGAAATCC
PpXKS1-NotI-R	ATAGCGGCCGCTTACTTCTCTAGAGTTTGTTC
GCW14p-F	ATATATGCATCAGGTGAACCCACCTAAC
GCW14p-R	TGGTAACCATCGTTTCTTTTGTTGTTGAGTGAAG
PpXKS1-F	AACAACAAAAGAAACGATGGTTACCAAAGAAATC
PpXKS1-R	ATATATATGCATGGATCCGCACAAACGAAG
GCW14p-2-F	ATATATGGATCCAGTGAGCTCGCTGGGTGA
GCW14p-2-R	TTACGGACATCGTTTCTTTTGTTGTTGAGTGAAGCGAG
PpIlv5-F	TCACTCAACAACAAAAGAAACGATGTCCGTAAGAAAT G
PpIlv3-R	ATATATGGATCCGCACAAACGAAG
**RT-PCR primers**	
PpIlv5-RT-F	GGTCAGAATTGCTTCTCAAGCCT
PpIlv5-RT-R	CGTGGACAACTTCTTCGACACC

PpIlv3-RT-F	CCGGTCAGATCACCGAGGAG
PpIlv3-RT-R	TCACCTCAGCACAGGATGCC
PpIlv6-T2A-PpIlv2_codon opt-RT-F	AAGGTGCTGGTCACATGGCT
PpIlv6-T2A-PpIlv2_codon opt-RT-R	GGAACACCATCAGCCAAAGCA
LlkivD-T2A-ScADH7-RT-F	TCGCAGAACAGAACAAATCC
LlkivD-T2A-ScADH7-RT-R	AAATACCGATGCCCTGAAAT
PpXKS-RT-F	TTCGAAGGGCGTTCATTGAC
PpXKS-RT-R	ATCTAACGCTTCGACCCACA
PspXI-RT-F	TGGAGCCAACTAAGCACCAA
PspXI-RT-R	TCAACAGCACAAGCCAACTC
LpXI-RT-F	TGTCTTGGTGGCACACTTTG
LpXI-RT-R	AGCCTTAGCCAACTCCATAGG
PpACT1-RT-F	ACAGTGTTCCCATCGGTCGT
PpACT1-RT-R	GGATTGAGCCTCGTCACCGA

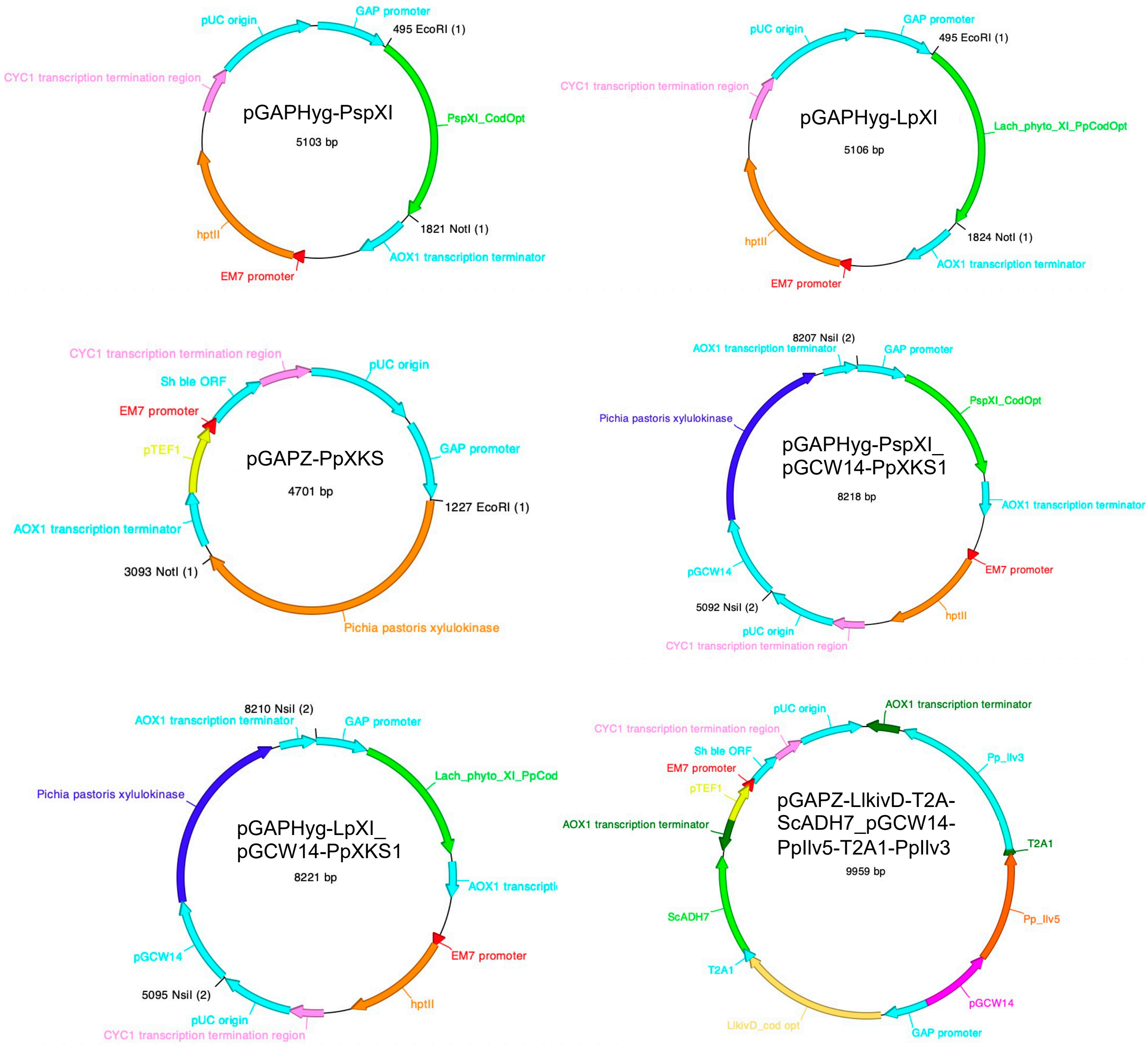

Figure S1. Plasmid maps for plasmids used in this study.

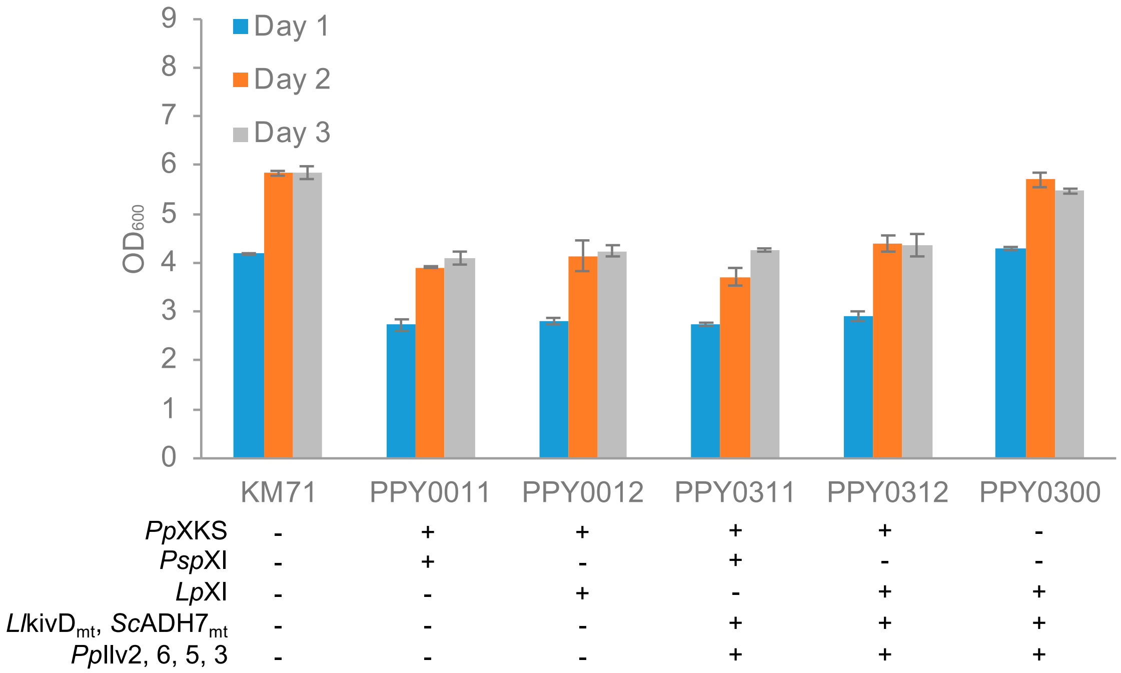

Figure S2. Growth of engineered *P. pastoris* in a mixed-carbon source medium. Engineered strains were precultured in 5-mL aliquots in MGYH minimal medium (2% glycerol) overnight and used to inoculate 5 mL yeast selective medium (2% total sugar; 1.70% glucose and 0.30% xylose) to achieve an initial optical density of 0.05 at 600 nm (OD_{600}). The cultures were grown at 30 °C and 250 rpm in an orbital shaking incubator. Samples were taken at 24, 48, and 72 hours for isobutanol quantification and OD_{600} measurement. Values are the mean of three biological replicates ± standard deviation (n = 3). Lp, *Lachnoclostridium phytofermentans*; Psp, *Piromyces* sp. E2; Pp, *Pichia pastoris*; Ll, *Lactococcus lactis*.
